# Different modality-specific mechanisms mediate serial dependence effects in visual and auditory perception

**DOI:** 10.1186/s12915-026-02515-9

**Published:** 2026-03-04

**Authors:** Irene Togoli, Michele Fornaciai, Domenica Bueti

**Affiliations:** 1 Life Sciences Department, University of Trieste, Trieste, Italy; 2 International School for Advanced Studies (SISSA), Trieste, Italy

**Keywords:** Perceptual history, Serial dependence, EEG, Time perception

## Abstract

**Background:**

Perceptual history plays an important role in sensory processing and decision making, shaping how we perceive and judge external objects and events. Indeed, past stimuli can bias what we are currently seeing in an attractive fashion, making a current stimulus appear more similar to a preceding one than it actually is. Such attractive “serial dependence” effects concern virtually every aspect of perception, suggesting that they may reflect a fundamental principle of brain processing. However, it is unclear whether the ubiquitous nature of serial dependence is due to an underlying centralised mechanism, or to the existence of separate mechanisms implemented independently in different perceptual pathways. Here we address this question by assessing the behavioural and neural signature of serial dependence in the auditory and visual sensory modality (in separate conditions), in the context of time perception.

**Results:**

Our results first show a double dissociation between the two modalities, whereby auditory serial dependence is selective for the features of the stimuli (i.e. reduced effect when successive stimuli have different features) but not their position, and vice versa in vision. Electroencephalography results further support a difference between the visual and auditory modality, demonstrating that the signature of serial dependence unfolds according to different dynamics in the two modalities.

**Conclusions:**

Overall, our results suggest that the serial dependence effect is mediated by different and at least partially independent modality-specific mechanisms, potentially based on the same computational principle implemented in different sensory pathways.

## Background

What we perceive at any given moment is not solely determined by the physical information reaching our sensory organs, but it is strongly influenced by the temporal context that such information is embedded in. Indeed, the recent history of stimulation, or perceptual history (i.e. information from the stimuli processed in the recent past) has been shown to shape how we perceive and judge stimuli in the external environment. One classic example of how a past stimulus can affect what we perceive in the present is the process of perceptual adaptation, whereby a long, sustained exposure to a stimulus causes the perception of the subsequent one to be strongly repulsed away from it [1]. Differently from adaptation, briefer exposures to sensory stimuli can induce attractive biases across successive stimuli, effectively making a stimulus appear more similar to its preceding one than it actually is [2]. This attractive influence, named *serial dependence*, has been shown to be ubiquitous in vision, spanning from basic perceptual dimensions such as orientation [2–4], duration [5] or numerosity [6, 7], to more complex attributes such as face identity [8]. Similar biases have been also demonstrated in auditory perception ([9–12]; see also [13, 14] for a similar effect in audio-visual timing), although evidence in this modality is more limited compared to visual perception.

While studies investigating serial dependence have progressively refined our understanding of its properties, pinpointing the exact nature of this effect and its neural mechanisms has proven to be difficult. Several ideas have been advanced so far to explain the underlying mechanisms of this phenomenon, proposing that serial dependence occurs either at the sensory/perceptual level (i.e. thus biasing directly what we perceive; [15, 16]), or at post-perceptual processing stages (i.e. thus biasing how we remember or judge a stimulus; [4, 17]). For instance, serial dependence has been interpreted as a signature of a perceptual mechanism supporting visual stability and continuity [16, 18–20]. Alternatively, the attractive effect has been linked to a higher-level mechanism based on decision-making and memory, depending on the *judgment* of past stimuli rather than the stimuli per se [4, 21, 22]. Both these frameworks are supported by different findings showing properties consistent with perception [15, 23–26] or effects consistent with higher-level post-perceptual processes [4, 22, 27–29], leaving the nature of serial dependence a highly debated topic. Electroencephalography studies show a neural signature of perceptual history at early latencies after the onset of a stimulus (i.e. 50–150 ms; [23, 24, 30]), emerging even in the absence of an explicit task [24]. This suggests that although serial dependence might *originate* at high-level stages, it *operates* during perceptual processing. To reconcile these different ideas and lines of evidence, a possibility is that serial dependence might emerge at different brain processing stages, both perceptual and post-perceptual [31]. Indeed, rather than reflecting the operation of a single mechanism, serial dependence might be supported by similar computations implemented independently at different levels of the processing hierarchy. Different mechanisms might thus be preferentially engaged by different paradigms more heavily relying on perceptual processes or memory and decision-making [31]. Moreover, the effect measured behaviourally is not necessarily the result of a single mechanism, but likely the end result of a combination of different processes occurring throughout the brain processing stream [32, 33].

An important feature of serial dependence that may help to understand its mechanisms is its widespread nature across several perceptual domains and sensory modalities. Considering this feature, our question is: does the emergence of the serial dependence bias involve a unique, generalised mechanism shared across dimensions and modalities, or does it involve a series of distinct mechanisms implemented independently in different sensory pathways? Previous results show a dissociation in the mechanisms of decisional carry-over effects in visual and auditory perception [12], as suggested by a different degree of selectivity for the features of the stimuli (frequency and shape). Results from Li et al. [12], however, show this dissociation only for the effect induced by past judgments, while past stimuli induced an opposite, repulsive effect lacking such selectivity. On the other hand, there is evidence that serial dependence, similarly to other phenomena like central tendency [34, 35] and perceptual adaptation [36, 37], can transfer across modalities [38], although the evidence is mixed [12, 39]. The existence of cross-modal serial dependence is a particularly interesting possibility, as it could suggest the existence of a supra-modal mechanism. However, the integration of signals from different modalities does not necessarily require a supra-modal mechanism and can occur via signals transmitted between early sensory cortices [40]. Whether perceptual serial dependence in different senses involves a generalised supra-modal mechanism or lower-level modality-specific mechanisms thus remains unclear.

Answering this question would allow to better discern the nature of the serial dependence phenomenon and its underlying neural mechanisms. If serial dependence is mediated by a single, centralised mechanism, then different effects (i.e. in different sensory modalities) measured with the same task should show consistent properties. Alternatively, if serial dependence involves at least partially dissociable mechanisms, effects occurring along different perceptual pathways should show unique, specific properties. To address these possibilities, we tested and compared the serial dependence effect and its neural (electrophysiological) signature in time perception, in two separate conditions involving either auditory or visual stimuli.

In Exp. 1, we investigated the properties of the behavioural serial dependence effect in auditory and visual perception, focusing on two key aspects: feature selectivity (i.e. whether the effect generalises across stimuli with different contextual features) and spatial selectivity (i.e. whether the effect extends across different spatial locations). Indeed, addressing the selectivity of the effect in different modalities should reveal the “rules” according to which serial dependence emerges, providing insights into the mechanism(s) underlying this phenomenon. What we know from previous results is that visual serial dependence is spatially selective [2, 7, 26], but not particularly selective for contextual features such as colour or even the structure of the stimuli [3, 39, 41]. Serial dependence seems thus to be selective for a limited set of stimulus characteristics, rather than requiring an exact match of the stimulus features. In auditory perception, while the spatial selectivity of the effect has never been tested, past stimulus information (or information about past decisions) has been shown to be encoded in a feature-selective fashion [12, 42].

According to our main hypothesis, if serial dependence entails a centralised mechanism downstream to the sensory processing pathways, we expect auditory and visual effects to show the same pattern of selectivity. Indeed, a shared centralised mechanism is expected to apply the same processes and computations to comparable signals, irrespective of the sensory modality they originate from. Alternatively, if sensory modality-specific mechanisms are involved in generating serial dependence, we would expect different patterns of selectivity specific to each modality. Different sensory modalities generally show different selectivity tailored to the nature of the signals processed. For instance, while vision has typically a fine spatial resolution but poor temporal resolution—explaining the spatial selectivity of the effect—auditory perception is instead much more sensitive to the temporal properties of a stimulus and less sensitive to its spatial properties [43–45]. Therefore, if serial dependence arises from sensory-specific mechanisms, we would expect an opposite pattern of selectivity compared to vision: a stronger selectivity for the (temporal) features of the stimuli, and weaker selectivity to their spatial position. This hypothesis is also supported by previous studies showing a different degree of feature selectivity of decisional carry-over effects in visual and auditory perception [12], with the latter being more selective for the features of the stimuli.

To overcome the intrinsic differences in visual and auditory features and the sensitivity of different sensory modalities (i.e. the visual modality being more sensitive to the spatial properties of the stimuli, while the auditory modality is more sensitive to the temporal properties), we chose stimulus features that are easily and immediately discriminable (i.e. colour for the visual stimuli, sound frequency [pitch] for the auditory stimuli), and large spatial differences that are easily discriminable also in auditory perception.

**Fig. 1:**
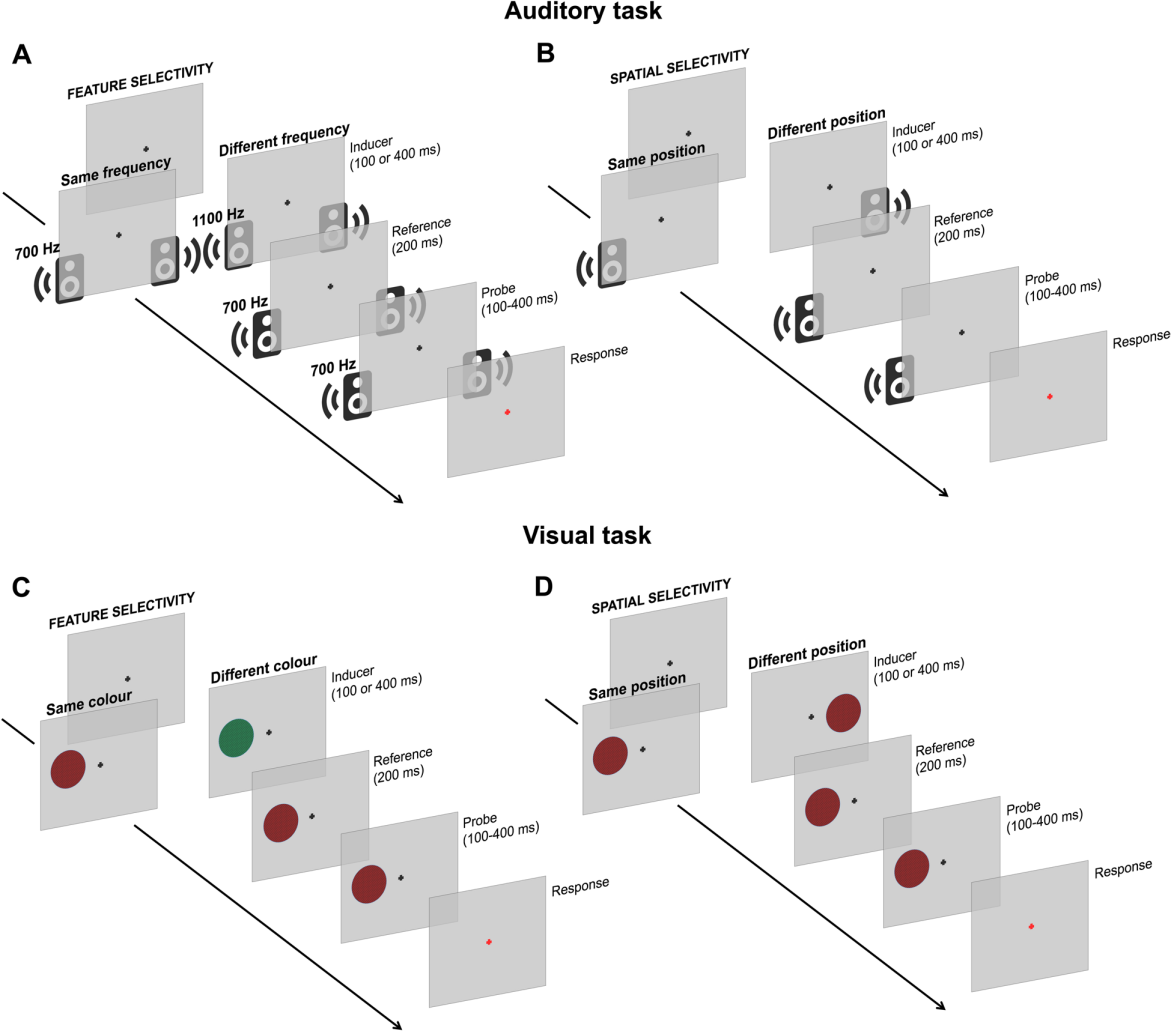
General paradigm employed in Exp. 1 and Exp. 2. In all conditions, each trial included the presentation of a task-irrelevant inducer (100 or 400 ms), followed by a reference (200 ms) and a probe stimulus (100–400 ms). Participants discriminated the reference and probe indicating which one seemed to last longer (duration discrimination task). **A** Procedure of the “feature-selectivity” condition in the auditory task. In this condition, the inducer and reference had either the same frequency or different frequencies. **B** Procedure of the “spatial-selectivity” condition in the auditory task. Inducer and reference could be played from either the same speaker (i.e. same spatial position) or different speakers. **C** Feature-selectivity and **D** spatial-selectivity condition of the visual task. Except for a few differences in the timing of the stimulus presentation, aimed at optimising the procedure for EEG recording and analysis, the paradigms of Exp. 1 and Exp. 2 were identical. In Exp. 1, the inter-stimulus interval (ISI) between the inducer and reference was 250–350 ms, while the reference-probe ISI was 350–450 ms. In Exp. 2, the ISI between the inducer and reference was 500–600 ms, while the reference-probe ISI was 800–900 ms. In both cases, the inter-trial interval was 300–400 ms. In all cases, the reference and probe stimuli always had consistent properties (i.e. same features, same positions). Stimuli are not depicted to scale

In Exp. 2, we use electroencephalography (EEG) in addition to psychophysical testing, in order to disentangle the neural signature of perceptual history in the two sensory modalities. Specifically, we aimed at addressing how the preceding stimulus modulates the brain responses to the current one, as a function of the features of the stimuli and their spatial position. To this aim, we employed the same paradigm of Exp. 1, in order to additionally replicate the behavioural effects and achieve more robust conclusions (Fig. 1). The study was not pre-registered.

## Results

### Experiment 1

To disentangle whether serial dependence is mediated by a generalised supra-modal mechanism or sensory modality-specific mechanisms, we first compared the properties of serial dependence in the two modalities, at the behavioural level. Namely, we assessed two important characteristics of the effect: its selectivity for the features of the stimuli (“feature-selectivity” condition) and for their spatial position (“spatial-selectivity” condition). Note that although the position of the stimuli could be considered a “feature,” henceforth we will refer to “feature-selectivity” when modulating auditory frequency and visual colour, and “spatial-selectivity” when modulating the position of the stimuli, as a naming convention. In Exp. 1, a total of 20 participants was included in the analysis.

Considering the specialisation of different senses for the temporal (auditory) or spatial (visual) characteristics of the stimuli, the idea of modality-specific mechanisms predicts different patterns of selectivity in the two modalities [2, 3, 7, 12, 26, 39, 42]. Conversely, a supra-modal mechanism predicts similar properties, since a generalised mechanism is expected to apply the same processes and rules to comparable signals irrespective of their origin.

In the experiment, participants compared the duration of a constant reference (200 ms) with a probe duration varying from trial to trial (100–400 ms). A task-irrelevant inducer stimulus (either 100 or 400 ms) was presented before the reference, to induce serial dependence. Since the inducer stimulus was always task-irrelevant, we focused only on the effect of the stimulus magnitude, as no explicit response was made on the previous stimulus that can alternatively be used in data analysis (see also 7, 23, 30, 33, for a similar analytical approach). This however does not mean that no decision was made on the inducer, since implicit perceptual decisions related to the representation of the stimulus are likely to occur irrespective of whether performing a task was required or not. From this discrimination paradigm, we computed the perceived duration of the reference (i.e. accuracy) as a function of the inducer and its properties. Serial dependence was then assessed by computing a “serial dependence effect index”, based on the normalised difference between the reference perceived duration when it was preceded by a 400-ms inducer versus a 100-ms inducer, turned into percentage ([400 ms – 100 ms/100 ms] × 100). This index reflects the extent to which the perceived duration of the reference differs as a function of the inducer duration.

**Fig. 2:**
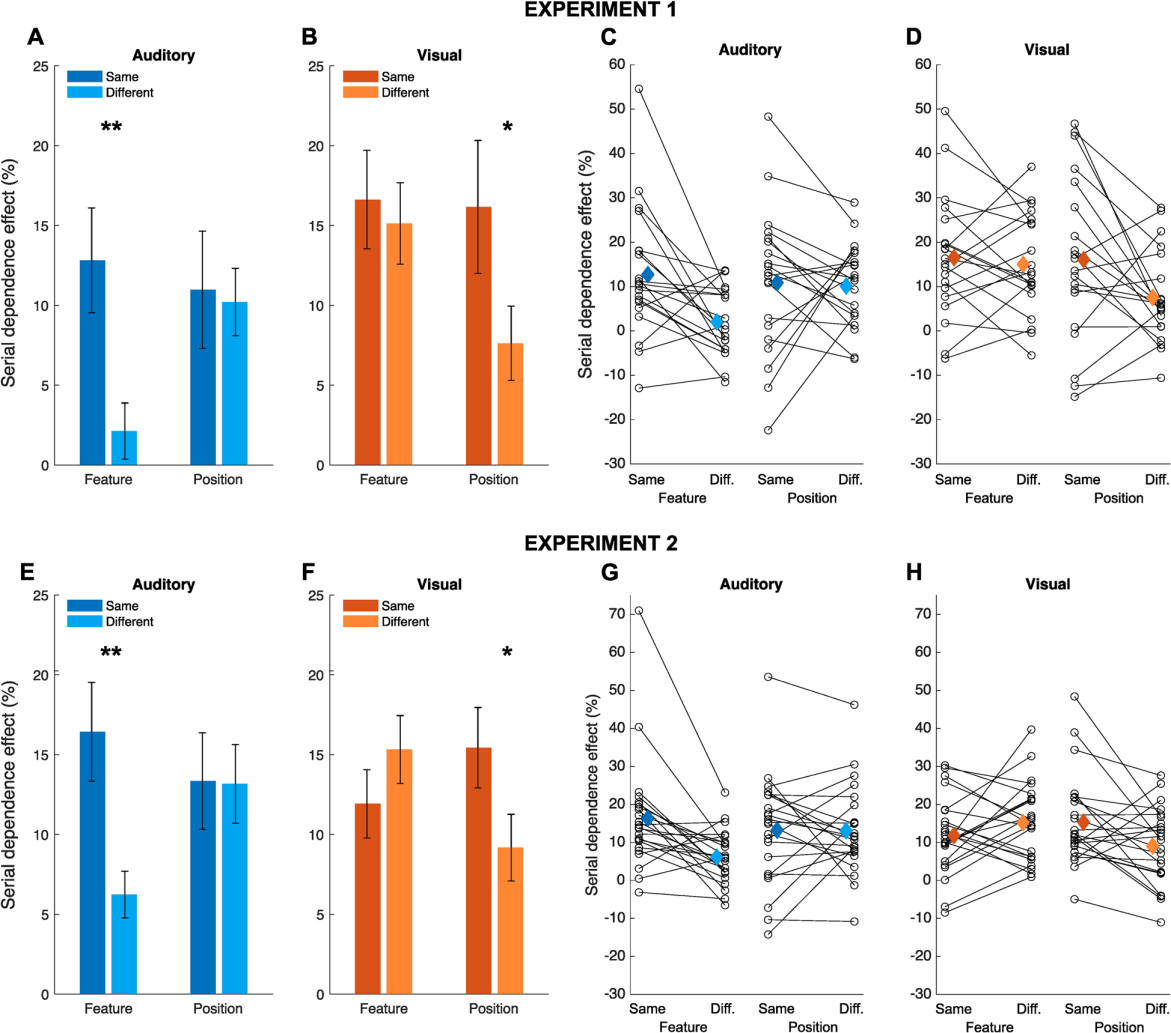
Behavioural serial dependence effects measured in Exp. 1 and Exp. 2. Average serial dependence effects measured in auditory (**A**) and visual perception (**B**), across the different conditions of Exp. 1 (feature-selectivity, spatial-selectivity). **C** Distribution of effects across the group, in the auditory condition. **D** Distribution of effects across the group, in the visual condition. Circles represent single subjects, while the diamonds show the average (same as **A** and **B**). **E** Average serial dependence effects measured in the auditory condition of Exp. 2. **F** Average serial dependence effects measured in the visual condition of Exp. 2. **G** Distribution of effects across the group tested in the auditory condition. **H** Distribution of effects across the group tested in the visual condition. Stars refer to the significance of paired *t*-tests: *p < 0.05, **p < 0.01. Error bars are SEM

The results of Exp. 1 are shown in Fig. 2A, B (average) and Fig. 2C, D (individual data). Overall, what we observed is a double dissociation between the properties of the effect in auditory and visual perception. Namely, the auditory effect was strongly selective for the features of the stimuli (Fig. 2A), on average dropping to almost zero when inducer and reference had different pitch, but did not show any spatial selectivity (i.e. identical effect irrespective of the spatial position of inducer and reference). Conversely, in vision (Fig. 2B), the effect did not show any selectivity for the features (i.e. colour) of the stimuli, while it showed instead a selectivity for their spatial position.

To assess the significance of these differences, we performed a linear mixed effect (LME) model regression including “modality” (visual vs. auditory), “condition” (feature vs. spatial selectivity), and “congruency” (same vs. different, referring to either features or position) as fixed effects, and the subjects as random effects. The results (*R*^2^ = 0.30) showed a significant three-way interaction between the different factors (beta = − 16.47, *t* = − 2.37, *p* = 0.019), which we followed up with a series of one-sample and paired *t*-tests.

First, with the exception of the auditory condition with different features (one-sample *t*-test against zero, *t(*19) = 1.21, *p* = 0.24; effect = 2.12%), all the effects in the individual conditions were significantly higher than zero (*t*(19) = 2.99–5.94, *p*-values < 0.008; effects spanning from 7.62% in visual/different positions condition to 16.62% in the visual/same features condition). Moreover, the selectivity of the bias was assessed by comparing the effect of stimuli having the same features or same position vs. stimuli having different features or presented in different positions. In auditory perception, we observed a significant difference in terms of the stimulus features, with a stronger effect when inducer and reference shared the same frequency (*t*(19) = 3.15, *p* = 0.005, Cohen’s *d* = 0.84). No difference was instead observed as a function of spatial position (*t*(19) = 0.21, *p* = 0.84, *d* = 0.05). The opposite pattern was observed in vision, with no significant difference according to the congruency of the features (*t*(19) = 0.54, *p* = 0.60, *d* = 0.11), but a significant difference according to the position of the stimuli (*t*(19) = 2.14, *p* = 0.045, *d* = 0.53). Taken together, these results show that serial dependence works differently in auditory and visual perception, following a pattern of selectivity specific for the tested sensory modality.

Besides the average effects in the different conditions tested, we also looked at the consistency of the effect at the individual level. Figure 2C and D show the distribution of effects across the group. In the auditory condition (Fig. 2C), the majority of participants showed a stronger serial dependence effect with similar stimulus features, with only a few participants showing the opposite trend. In the spatial selectivity condition, instead, the direction of the effects was less consistent and more mixed, with 13 participants showing stronger effects when the stimuli were in the same position, and 7 showing instead relatively large differences in the opposite direction (i.e. stronger effects with different positions), leading to similar average effects. In the visual modality (Fig. 2D), the feature selectivity condition yielded a roughly equal number of participants showing a stronger effect with the same stimulus features as with different stimulus features, resulting in similar average effects. On the other hand, in the spatial selectivity condition, stimuli in similar spatial locations yielded overall stronger effects compared to different locations, although several participants showed little difference across the two conditions. A lack of selectivity seems thus to be driven by a higher proportion of participants showing stronger effects when features or positions are different.

Finally, we tested whether effects measured in different conditions correlate with each other. Within the same modality, we focused on the conditions leading to the stronger effects, that is, the effects observed with similar features and similar spatial positions, and tested their relationship. However, no significant correlation was observed (auditory: *r* = 0.31, *p* = 0.18; visual: *r* = 0.36, *p* = 0.12). Similarly, no correlation was observed across different sensory modalities (auditory and visual same features condition: *r* = 0.14, *p* = 0.54; auditory and visual same positions: *r* = 0.26, *p* = 0.26).

### Experiment 2

In Exp. 2, we used an identical paradigm compared to Exp. 1, but with the addition of electroencephalography (EEG) aiming at capturing and comparing the neural signature of serial dependence in auditory and visual perception. Using the same paradigm further allowed us to replicate the findings of Exp. 1 in an independent sample, ensuring the robustness of the pattern of results. Differently from the within-subject design of Exp. 1, here two independent groups of participants performed the feature-selectivity and spatial-selectivity conditions. Two groups of 23 participants each were included in the two conditions.

First, the behavioural results of Exp. 2 (Fig. 2E, F) replicated the findings of Exp. 1. A linear mixed effect model including condition (between-subject factor), congruency, and modality as fixed effects, and the subjects as random effect (*R*^2^ = 0.37) showed again a significant three-way interaction between the factors (beta = − 19.68, *t* = − 3.64, *p* < 0.001). This was followed up with individual one-sample *t*-tests and paired *t*-tests. The results of one-sample *t*-tests showed significant serial dependence effects across all the conditions (*t*(22) = 4.27–7.21, all *p*-values < 0.001). The results of paired *t*-tests comparing the effect across in the same features/position vs. different features/position showed an identical pattern compared to Exp. 1. Namely, in the auditory modality (Fig. 2E), serial dependence was significantly stronger when inducer and reference shared the same frequency compared to when they had a different frequency (*t*(22) = 3.65, *p* = 0.001, *d* = 0.79). No significant difference was observed when the stimuli were presented in the same or in a different position (*t*(22) = 0.07, *p* = 0.94, *d* = 0.01). The opposite was true in vision (Fig. 2F), where serial dependence did not significantly differ based on the features of the stimuli (same vs. different; *t*(22) = − 1.35, *p* = 0.19, *d* = 0.33), but it was instead significantly stronger when inducer and reference were presented in the same position (*t*(22) = 2.39, *p* = 0.025, *d* = 0.56).

In terms of individual effects (Fig. 2G, H), we observed a similar pattern compared to Exp. 1, with the specific type of selectivity emerging according to the consistency of effects across the group. Namely, also in this case, the lack of selectivity seems determined by a higher proportion of participants showing stronger effects when the features or position of the stimuli was different.

Additionally, since we did not want to confuse the lack of auditory spatial selectivity with a trivial inability to discern the different locations of the auditory stimuli (i.e. considering that auditory spatial selectivity is notoriously poorer compared to vision; [43]), we also tested the ability of the participants to discriminate the location of the sound. In a brief testing session preceding the main experiment, sounds were presented to participants either from the left or the right speaker and their task was to identify its spatial location. In doing so, we confirmed that the different positions were clearly and consistently discriminable (accuracy ~ 99%). This suggests that the lack of spatial selectivity of the auditory serial dependence effect was not due to the inability of participants to discriminate the different locations of the stimuli.

#### EEG results—feature-selectivity condition

**Fig. 3:**
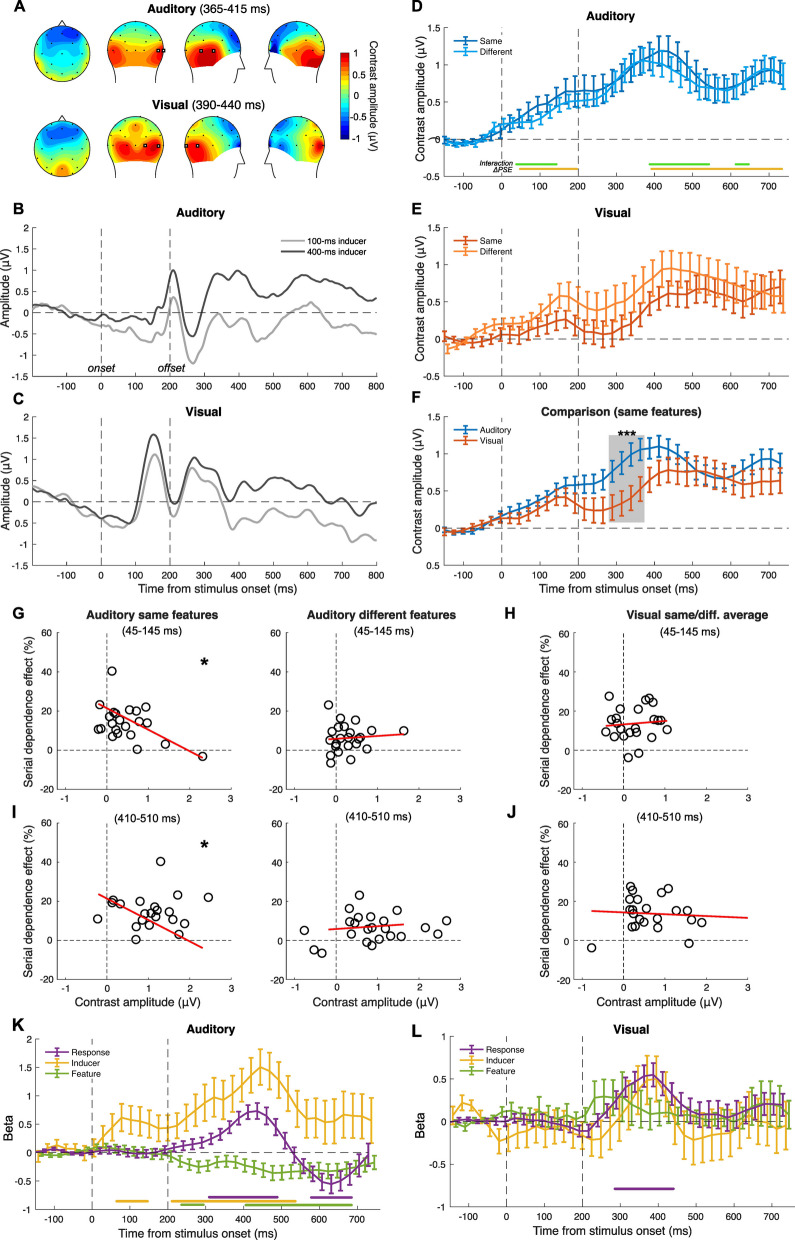
EEG results of the feature-selectivity condition of Exp. 2. **A** Topographic plots of activity, showing a 50-ms time window around the peak observed within each modality, used to select the target channels for further analyses (T8 and P8 in the auditory condition, P8 and PO4 in the visual condition; highlighted with bold markers in the figure). **B** Auditory ERPs time-locked to the onset of the reference, plotted separately as a function of the inducer duration. **C** Visual ERPs time-locked to the reference presentation. **D** Contrast waves in the auditory modality. The yellow and green lines at the bottom of the plot indicate the significant clusters of time windows observed in the LME analysis. **E** Contrast waves in the visual modality. **F** Comparison of the contrast waves obtained in the auditory and visual modality, averaged across the same and different features condition. The shaded area highlights the time window showing a significant difference between auditory and visual perception. The stars refer to the significance of a cluster-based non-parametric test, ***p < 0.001. Error bars are SEM. **G** Auditory condition: correlation between the serial dependence effect and the average contrast amplitude at 45–145 ms, when the stimuli had the same (left panel) or different (right panel) features. **H** Visual condition: correlation between serial dependence and the average contrast amplitude at 45–145 ms, computed from the average of the same and different features condition. **I** Auditory condition: correlation between the serial dependence effect and the average contrast amplitude at 410–510 ms. **J** Visual condition: correlation between serial dependence and the average contrast amplitude at 410–510 ms. **K** Results of the non-linear regression analysis in the auditory modality. Stars in **G** and **I** refer to the significance of correlation tests; *p < 0.05. **L** Results of the non-linear regression analysis in the visual modality. The lines at the bottom of each panel indicate the significant clusters observed in the analysis, with the colour matching that of the main plots. Error bars are SEM

Regarding the EEG results of the feature-selectivity condition (Fig. 3), we first identified a set of target channels to perform further analyses. Since it is difficult to come up with an a-priori selection of the target channels due to the lack of previous literature (especially when it comes to the neural correlates of auditory serial dependence), we took a data-driven approach. Namely, we computed the difference in the ERP amplitude relative to the reference stimulus as a function of the duration of the preceding inducer, irrespective of its features, and plotted its topographic distribution (see Fig. 3A). We then chose two channels (to increase the signal-to-noise ratio) based on the peak amplitude. Channels showing activity modulated by the inducer are indeed also expected to be sensitive to other manipulations that affect serial dependence, like having similar or different stimulus features. Based on this procedure, we selected the channels P8 and T8 in the auditory modality (peak = 1.21 µV at 390 ms after stimulus onset). In vision, the contrast amplitudes showed a more posterior topography, peaking at channels P8 and PO4 (0.87 µV, 414 ms). The peak at these two channels was observed at a similar latency compared to the auditory condition. Figure 3B and C show the average ERPs across the selected channels, time-locked to the onset of the reference and sorted according to the inducer duration.

To better assess the neural signature of serial dependence, we focused on the difference (“contrast”) in ERP amplitude as a function of the inducer duration, measured according to whether inducer and reference had the same or different features. The contrast reflects the extent to which the brain’s responses to the reference are affected by the preceding inducer, making it an ideal measure to assess the selectivity of the effect. To increase the signal-to-noise ratio, the contrast amplitude was computed across a series of 100-ms windows (with 25-ms step), in a sliding-window average fashion. Multiple comparisons were controlled with a cluster-based non-parametric (permutation) test. Since this approach is usually very sensitive [46], we also set a cluster threshold of at least three consecutive significant tests to make it more conservative. The contrast amplitude in auditory perception (Fig. 3D) showed a ramping pattern starting from stimulus onset and peaking at around 370–410 ms (1.18 µV and 1.05 µV, respectively, for the same and different features condition). No significant difference was observed as a function of the features of the stimuli (paired *t*-tests across conditions, max *t*(22) = 1.12, min *p* = 0.10). In vision (Fig. 3E), the contrast showed an earlier peak at 150–170 ms (0.25 µV and 0.60 µV for the same and different features condition), followed by a later, higher peak at around 450–510 ms (0.65 µV and 0.94 µV). Also in this case, we did not observe significant differences (i.e. clusters below the size threshold). Interestingly, the contrast amplitude in vision seemed higher (although not significantly) when inducer and reference had different features compared to when they were similar, potentially in line with the slightly stronger behavioural serial dependence effect observed in this condition (see Fig. 2F).

To characterise the link between the behavioural and neural signature of serial dependence, we first used a linear mixed-effect (LME) regression model performed across each time window within the reference epoch. Namely, we assessed the relationship between the behavioural (difference in PSE as a function of the inducer; ΔPSE = PSE_400ms_ – PSE_100ms_) and neural effects (amplitude of the contrast wave; ΔERP = ERP_400ms_ – ERP_100ms_). The features (same vs. different) of the stimuli represented an additional fixed effect, and the subjects the random effect (ΔPSE ~ ΔERP × Features + (1|subj)). Again, to control for multiple comparisons, we used a non-parametric cluster-based test (see *Methods*).

In the auditory modality, we observed two main significant latency windows for the effect of ΔERP: one early cluster spanning from 50 to 200 ms (*t* ≥ − 2.29, *p* ≤ 0.02), and a later one spanning from 380 to 730 ms (*t* ≥ −2.27, *p* ≤ 0.03). Moreover, we observed three latency windows in which the interaction between ΔERP and the features of the stimuli could predict the serial dependence effect: 40–140 ms (*t* ≥ 2.21, *p* ≤ 0.03, cluster *p* < 0.001), 380–540 ms (*t* ≥ 2.13, *p* ≤ 0.04), and 610–650 ms (*t* ≥ 2.07, *p* ≤ 0.044). All *p*-values from the non-parametric cluster-based tests were < 0.001. However, in vision, we did not observe any significant relationship between the behavioural and neural effect, nor a significant interaction between the ΔERP and the features of the stimuli. These results show that at least in auditory perception, the strength of the serial dependence effect is significantly related to the impact that the inducer has on auditory evoked potentials. Comparing the average contrast amplitude across the two modalities (Fig. 3F) showed overall lower amplitudes in vision and a significant difference between modalities at 280–360 ms (paired *t*-tests, *t* ≥ 1.83, *p* ≤ 0.01).

To better understand the relationship between serial dependence and the contrast amplitude observed in the LME (at least in the auditory condition), we performed additional analyses within two latency windows. Namely, we considered two 100-ms time windows, centred on the middle of the clusters showing a significant interaction in the LME analysis (see Fig. 3D): 45–145 ms and 410–510 ms. In the auditory modality, the early window (45–145 ms; Fig. 3G) showed a significant negative correlation between behavioural and neural measures when the stimulus features were similar (*r* = − 0.43, *p* = 0.043) but not when they were different (*r* = 0.08, *p* = 0.70). This is indeed in line with the interaction observed in the regression analysis. For consistency, we performed a similar analysis also in the visual condition (Fig. 3H), limited to the average between same and different features (as no interaction was observed). No significant correlation was observed (*r* = 0.09, *p* = 0.67). A similar pattern of results was observed in the later window (410–510 ms). That is, a significant correlation when the auditory features were similar (*r* = − 0.45, *p* = 0.030) but not when they were different (*r* = 0.17, *p* = 0. 42; Fig. 3I), and no correlation in the visual condition (*r* = − 0.09, *p* = 0.66; Fig. 3J).

Finally, we performed a non-linear regression analysis in order to potentially capture different effects that cannot be captured with a linear model. The non-linear regression allows us to use single-trial EEG data rather than aggregated ERPs and to focus on the trial-by-trial modulation of the different parameters as well as the binary response in the discrimination task. This can potentially provide a more sensitive index of the neural correlates of serial dependence and the selectivity of the effect. Specifically, we used the EEG activity in each trial (averaged across a series of 100-ms windows with a 25-ms step) as the dependent variable. As predictors, we entered the response in each trial (0 or 1, corresponding to “reference longer” or “probe longer” as a binary numerical predictor), the duration of the inducer (100 ms or 400 ms), and the features of the stimuli (same or different). Note that while the inducer duration and the features of the stimuli reflect properties that are already evident at the onset of the reference, the response will only occur much later in the trial, after the presentation of the probe stimulus.

In the auditory condition, in terms of the influence of the inducer on EEG responses (Fig. 3K), we observed two main clusters of significant time windows: 70–140 ms (one-sample *t*-tests and cluster-based non-parametric tests; *t* ≥ 2.09, *p* ≤ 0.02, cluster *p* = 0.006), and 220–530 ms (*t* ≥ 2.14, *p* ≤ 0.044, cluster *p* < 0.001). Regarding the influence of the stimulus features, we observed significant effects at 240–290 ms (*t* ≥ − 2.19, *p* ≤ 0.04, cluster *p* < 0.001) and 410–680 ms (*t* ≥ − 2.19, *p* ≤ 0.04, cluster *p* < 0.001). Finally, we observed a significant relationship between EEG activity and the behavioural response at 310–490 ms (*t* ≥ 2.51, *p* ≤ 0.02, cluster *p* < 0.001), and 580–680 ms (*t* ≥ − 2.10, *p* ≤ 0.047, cluster *p* < 0.001). In the visual condition (Fig. 3L), we did not observe any consistent effect of the inducer duration and the congruency in the features of the stimuli. However, we found a significant effect of the response, at 290–430 ms (*t* ≥ 2.45, *p* ≤ 0.02, cluster *p* < 0.001). The effect of the inducer showed a peak coincident with the effect of the response, but it did not reach statistical significance.

#### EEG results—spatial-selectivity condition

After assessing the properties of the neural signature of serial dependence in the feature-selectivity condition and its relationship with the behavioural effect, we went on and performed a similar set of analyses on the data from the spatial-selectivity condition.

**Fig. 4:**
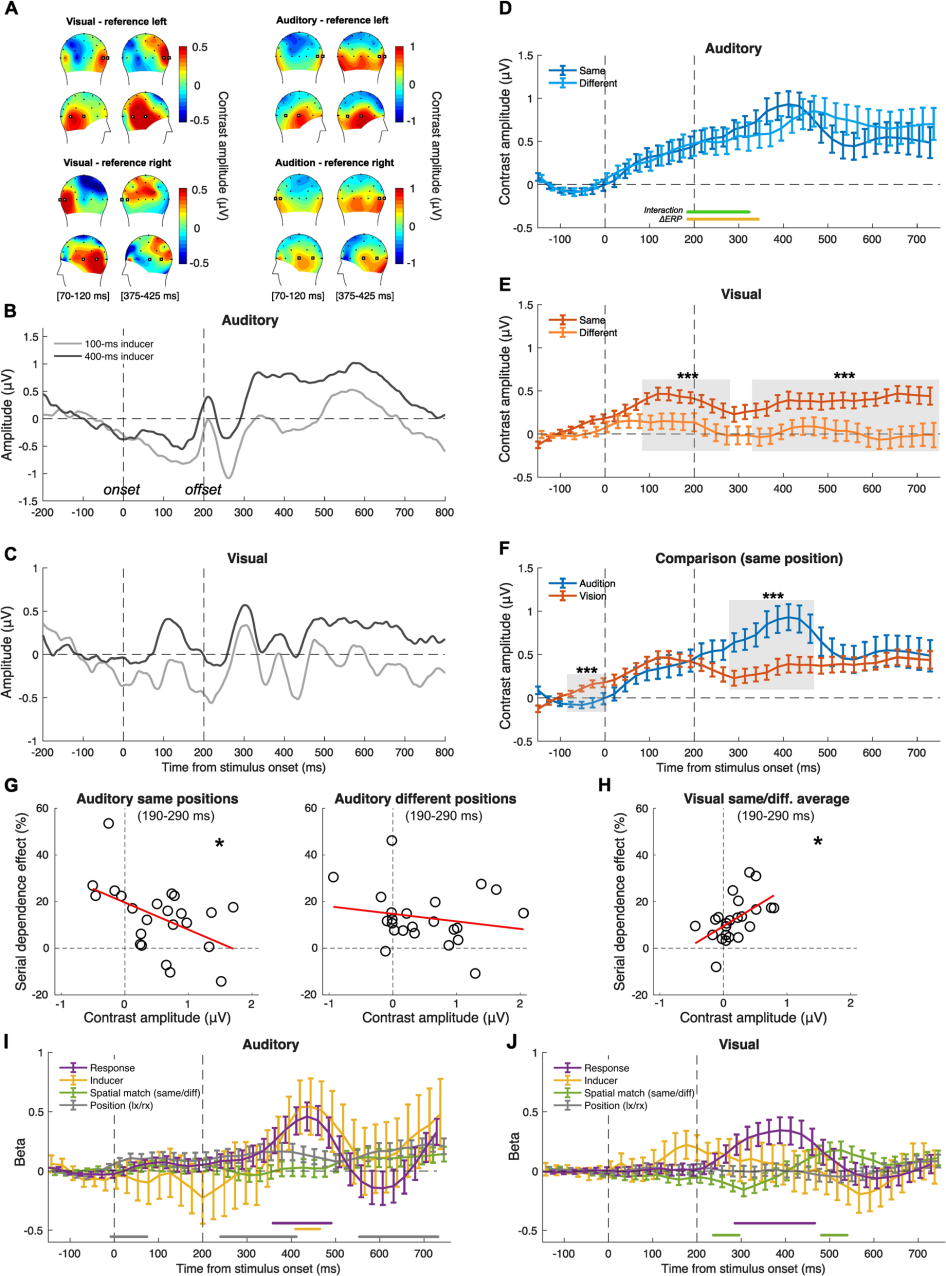
EEG results of the spatial-selectivity condition of Exp. 2. **A** Topographic plots of activity across the scalp, in two 50-ms time windows around the peaks observed in the visual (70–120 ms) and auditory (375–425 ms) modality, which were used to select the target channels for further analyses (T8 and P8 in the right hemisphere, T7 and P7 in the left hemisphere; highlighted in the figure with bold markers). **B** Auditory ERPs time-locked to the onset of the reference. **C** Visual ERPs time-locked to the reference presentation. **D** Contrast waves in the auditory modality (same and different positions). The yellow and green lines at the bottom of the plot indicate the significant clusters of time windows observed in the LME analysis. **E** Contrast waves in the visual modality. **F** Comparison of the contrast waves obtained in the auditory and visual modality, averaged across the same and different position condition. The shaded area highlights the time window showing a significant difference between auditory and visual perception. The stars refer to the significance of a cluster-based non-parametric test. Error bars are SEM. ***p < 0.001. **G** Auditory condition: correlation between the serial dependence effect and the average contrast amplitude at 190–290 ms, when the stimuli had the same (left panel) or different (right panel) position. **H** Visual condition: correlation between serial dependence and the average contrast amplitude at 190–290 ms, computed from the average of the same and different position. Stars in **G** and **H** refer to the significance of correlation tests; *p < 0.05. **I** Results of the non-linear regression analysis in the auditory modality. **J** Results of the regression analysis in the visual modality. The lines at the bottom of each panel indicate the significant clusters observed in the analysis, with the colour matching that of the main plots. Error bars are SEM

First, in the spatial-selectivity condition, ERPs were computed in a slightly different way to account for the different positions of the stimuli. Namely, the ERPs were computed as the average of the channels contralateral to the reference stimulus positions: T7 and P7 in the left hemisphere (reference presented on the right); T8 and P8 in the right hemisphere (reference presented on the left). The topographic distribution of activity seemed indeed to be localised to electrodes contralateral to the reference stimulus, especially in the visual condition (Fig. 4A). In the auditory modality, this lateralisation was less evident, but we implemented the same strategy for consistency. The overall peak of activity in the visual modality was observed at 95 ms after stimulus onset (0.45 µV), and at 400 ms (0.71 µV) in the auditory modality. Figure 4B and C show examples of the ERPs time-locked to the reference stimulus, sorted according to the duration of the inducer stimulus (100 and 400 ms) but irrespective of the position of the stimuli.

Similarly to the feature-selectivity condition, here we focused on the contrast between different inducer durations according to the position of the inducer and reference stimuli (same vs. different; Fig. 4D–F). In the auditory modality (Fig. 4D), we observed activity increasing from stimulus onset up until around 400 ms. Comparing the same vs. different condition with paired *t*-tests showed no significant difference across them (max *t*(22) = 1.21, min *p* = 0.22). In vision, instead, we observed a much stronger effect of the inducer when it was presented in the same position as the reference. A series of paired *t*-tests (complemented with a cluster-based non-parametric test) showed significant effects spanning almost the entire epoch: 90–270 ms (*t* ≥ 2.11, *p* ≤ 0.04, cluster *p* < 0.001), and 340–750 ms (*t* ≥ 2.17, *p* ≤ 0.048, cluster *p* < 0.001).

To address the link between the behavioural and neural signature of perceptual history, we performed again a LME regression analysis at each time window (100 ms each, 25-ms step) throughout the epoch (ΔPSE ~ ΔERP × Position + (1|subj)). In the auditory modality (Fig. 4D), we observed a significant effect of ΔERP spanning 190–340 ms (*t* ≥ − 2.37, *p* ≤ 0.02). Overlapping with this cluster, we also observed a significant interaction between ΔERP and the positions of the stimuli (190–320 ms, *t* ≥ 2.04, *p* ≤ 0.047). On the other hand, in vision (Fig. 4E), we did not observe any significant effect, similarly to the results of the feature-selectivity condition. Finally, comparing the contrast waves of the two sensory modalities against each other (limited to the case where inducer and reference were presented in the same position; Fig. 4F) showed a significant difference at 290–460 ms (*t* ≥ − 2.18, *p* ≤ 0.03), and an earlier and smaller cluster before stimulus onset, from − 80 to 0 ms (*t* ≥ 2.17, *p* ≤ 0.03). All *p*-values from the cluster-based tests were < 0.001.

In this case, additional analyses were performed in a window spanning 190–290 ms, centred to the middle of the cluster showing a significant interaction in the LME (see Fig. 4D). In the auditory modality (Fig. 4G), we observed a significant negative correlation between the serial dependence effect and contrast amplitude when the positions were the same (*r* = − 0.49, *p* = 0.018) but not when they were different (*r* = − 0.19, *p* = 0.38). Interestingly, when considering the average of the same and different positions in the visual condition, we found a significant correlation in the opposite, positive direction (Fig. 4H; *r* = 0.54, *p* = 0.008).

Finally, we performed a non-linear regression analysis. As predictors, we used the inducer duration (100 vs. 400 ms), the position of the stimuli on the screen (left vs. right hemifield), the spatial match of inducer and reference position (same vs. different), and the response that the participant will provide at the end of the trial (0 or 1, i.e. “reference longer” or “probe longer”). The results of this analysis are shown in Fig. 4I, J. In the auditory modality (Fig. 4I), we found a significant relationship between EEG amplitude and the participants’ response in the task, at 360–490 ms (*t* ≥ 2.41, *p* ≤ 0.02). The later negative deflection in beta values however did not reach statistical significance in this condition. Besides that, we observed an effect of the inducer duration at around 410–460 ms (*t* ≥ 2.08, *p* ≤ 0.049). While the spatial match between inducer and reference had no significant effect, we observed a small but significant influence of position (left vs. right), which was unexpected. This effect indicates that EEG amplitude differed slightly, but systematically, according to the position of the reference irrespective of whether it was consistent with the inducer or not. The effect of position was evident across three clusters, spanning –5 to 70 ms (*t* ≥ 2.31, *p* ≤ 0.03), 240–410 ms (*t* ≥ 2.09, *p* ≤ 0.048), and 570–730 ms (*t* ≥ 2.30, *p* ≤ 0.03).

In the visual modality (Fig. 4J), we observed again a significant relationship between EEG and behavioural responses, in a time window spanning from 290 to 460 ms (*t* ≥ 2.20, *p* ≤ 0.04). In line with the spatial selectivity of the effect, we also observed a significant effect of spatial match at 240–290 ms (*t* ≥ − 2.15, *p* ≤ 0.04), and 480–530 ms (*t* ≥ 2.18, *p* ≤ 0.04). All the cluster-based non-parametric tests in this context showed a *p*-value < 0.001.

## Discussion

In this study, we aimed at investigating the mechanisms underlying serial dependence in different sensory modalities. Evidence so far shows that serial dependence is ubiquitous in vision [2, 7, 8, 47], and attractive effects have also been reported in auditory perception [9–11] and across different modalities [38]. Considering this seemingly generalised influence of serial dependence, our question is as follows: is this phenomenon mediated by a common, perhaps centralised (i.e. supra-modal), processing mechanism? Or is it the result of similar computations implemented independently across different perceptual pathways?

Previous work demonstrated that serial dependence in different perceptual dimensions shows different properties. For example, effects in orientation perception are usually tuned to the similarity of successive stimuli and are weakly spatially selective [2, 26]. Instead, effects in numerosity perception are less tuned and more tightly localised [7, 41, 48]. Whether such differences truly reflect distinct mechanisms is unclear, as effects in different domains are typically measured with very different paradigms and tasks. Here we thus aimed at testing a possible dissociation of the effects while keeping the testing procedures as similar as possible. To this aim, we focused on the difference between visual and auditory perception, which have clearly dissociable sensory processing streams. We specifically chose time (i.e. duration) perception, since interval duration can similarly be delivered as a visual or an auditory input. Then, our main manipulations aimed at testing the feature and spatial selectivity of the effect in these two modalities. Although auditory frequency and visual colour are not directly comparable, we chose these features as they represent two “primary”, fundamental dimensions of the respective sensory modality. In terms of spatial selectivity, on the other hand, we made sure to have a large enough spatial difference to be immediately discriminable also in auditory perception. Our results, overall, provide evidence that serial dependence in visual and auditory perception is mediated by different mechanisms, by demonstrating that (a) serial dependence works differently in different sensory modalities, showing different patterns of selectivity, and that (b) it shows at least partially different neural signatures in different modalities.

First, the behavioural results of Exp. 1 and Exp. 2 show a double dissociation in the properties of serial dependence. Namely, auditory serial dependence shows selectivity for the features of the stimuli (i.e. sound frequency, or pitch), yielding a stronger effect when successive stimuli are similar to each other. However, it is not very selective for the position of the stimuli, even if they are presented in clearly different and discriminable positions. Conversely, vision does not show selectivity for the features (i.e. colour) of the stimuli, while it instead shows a significantly stronger effect when the stimuli are presented in the same position. Interestingly, these results are consistent with the “specialisation” of different sensory modalities to different perceptual dimensions: higher spatial resolution but low temporal resolution in vision, and vice versa in auditory perception (e.g. see for instance [43, 49, 50]). Regarding visual serial dependence, some effect could be observed even when the stimuli are presented in different positions, consistently with the relatively broad spatial selectivity of the effect shown in previous studies [2, 7, 26]—potentially suggesting the involvement of very large receptive fields or feedback signals from mid- or high-level areas [33, 51]. These findings are also in line with Li et al.’s [12] results, which suggested the existence of sensory modality-specific mechanisms for attractive decisional carry-over effects (i.e. serial dependence based on past decisions). Indeed, Li et al. observed different patterns of feature selectivity in the visual and auditory modality, similarly to our results. However, Li and colleagues observed attractive effects only by previous judgements, while previous stimuli induced repulsive effects lacking such selectivity. Our results further suggest that feature selectivity and a difference across modalities can also be observed in the absence of explicit responses made on past stimuli. This does not mean that what induced serial dependence in this instance is necessarily the physical stimulus per se (that is, the low-level sensory information delivered by the inducer). Even if no explicit judgment or response was required for the inducer stimulus, implicit perceptual decisions are expected to occur irrespective of the task, related for instance to the perceptual representation of the stimulus (i.e. its perceived magnitude). Attractive serial dependence effects can thus be induced by previous perceptual decisions rather than the previous stimuli themselves—which is in fact likely considering that serial dependence has been shown to operate according to the perceived rather than physical properties of a stimulus [51]. Different patterns of selectivity might thus arise from a different interaction between stimulus-driven repulsive effects and decision-driven attractive effects [4, 17, 30, 52, 53]. Since our paradigm did not include any explicit judgment made on the inducer stimuli, this is however difficult to assess since we do not have a clear measure of perceptual decisions made on the inducer (its perceived magnitude, for instance), and therefore it remains an open question.

While the effects across Exp. 1 and Exp. 2 are largely identical, one difference is that in Exp. 2, the visual feature selectivity condition showed a stronger effect with different features, albeit the comparison was not significant. Although interesting to note, it is difficult to interpret this difference, and whether it potentially reflects some methodological differences across the experiments (i.e. different ISIs) or the variability of the effect remains unclear.

Looking at the individual data (see Fig. 2C, D and G, H) shows that the effect is quite variable across the group. Interestingly, in conditions lacking selectivity, several participants actually show increased effects when the features or positions of the stimuli are different. However, our study was not designed to assess effects at the individual level. Thus, whether this increased bias for different features/positions reflects a functional aspect of serial dependence, the noisiness of the data, or the natural variability of the effect remains unclear. Due to this, we keep our interpretation focused on the average effect. Still, this represents a potentially interesting aspect that future studies should address with dedicated experiments, focusing more closely on the individual subjects. Finally, our data also show no correlation in the effects across modalities. However, it does not show a correlation also *within* each modality, suggesting that this analysis is likely limited by a lack of power (i.e. relatively small sample size). No conclusion could thus be drawn from this lack of correlation.

Regarding the EEG results, our findings show different patterns of activity that support the idea of different modality-specific mechanisms. As a first signature of serial dependence, we considered the contrast (i.e. difference) between the ERPs evoked by the reference when it was preceded by the long (400 ms) versus the short (100 ms) inducer. This provided a measure of the impact of serial dependence on brain responses to the reference stimulus.

First, in both modalities, the contrast amplitude starts to ramp up very early after stimulus onset, suggesting that serial dependence affects the earliest stages of sensory processing (in line with previous EEG studies [23, 33, 54]). Second, however, the contrast amplitude unfolds differently in time, showing different peaks and a different overall strength. Specifically, while in the auditory condition the amplitude increases up until a peak at around 400 ms, the visual modality shows a much earlier peak (~ 150 ms). After its initial peak, the amplitude in vision tends to decrease, leading to a significant difference compared to the auditory stimuli at around 300–400 ms. This pattern might in turn suggest a difference in how past stimulus information is propagated during stimulus processing, with a faster integration of past information during visual processing. Interestingly, while the spatial selectivity of the effect is well captured by the difference in contrast amplitude in the visual condition (Fig. 4E), the feature selectivity of the auditory effect (Fig. 3E) does not seem to be reflected by the contrast amplitude. This shows that in auditory perception, a mismatch in the stimulus features does not necessarily prevent or reduce the encoding of past information like a difference in position does in vision, suggesting an additional difference in how the selectivity is achieved in different modalities. Spatial selectivity could for instance arise from a localised encoding of stimulus information. Feature selectivity in auditory perception, instead, might stem from a gating mechanism preventing the influence of perceptual history when the past stimulus does not match the current one (see also [30] for a similar interpretation of the effects across different dimensions).

The results of the linear regression analysis further show a significant link between the contrast amplitude measured with EEG and the behavioural effect, at least in the auditory condition. In the feature-selectivity condition, the auditory contrast amplitude can successfully predict the behavioural effect at early latencies, starting at around 50 ms after stimulus onset. In the spatial-selectivity condition, instead, this significant relationship seems to emerge right after the offset of the stimulus. This in turn suggests a difference in how the influence of perceptual history unfolds over time as a function of how the stimuli are manipulated. A possibility explaining this difference may be the predictability of the sequence of events in the two conditions. Namely, while in the feature-selectivity condition there was no uncertainty about the properties of the reference stimulus—which was always the same—the position of the reference in the spatial-selectivity condition was randomised. Thus, with more predictable stimuli, it is possible that serial dependence could start to play its role at a much earlier stage compared to less predictable stimuli.

On the other hand, in the visual modality, the linear regression model did not yield any significant effect in the feature-selectivity and spatial-selectivity condition, despite the contrast amplitude showing a clear influence of the inducer on the ERPs evoked by the reference. This possibly reflects another difference across the two modalities: while the behavioural and neural signature of serial dependence show a more linear relationship in auditory perception, visual signals do not show such a clear relationship. If a shared mechanism was involved in generating serial dependence, then we would have expected to find comparable results across modalities. However, caution is in order when interpreting this difference, since the lack of significant results in the visual condition might also reflect a lower signal-to-noise ratio. Vision is indeed expected to be less sensitive to duration compared to auditory perception (see for instance [30]), leading to noisier brain processing and perceptual representations. This is also suggested by the lower contrast amplitudes observed in vision compared to the auditory modality (see Figs. 3F and 4F), potentially reflecting a lower sensitivity. Thus, different signal-to-noise ratio levels across the two modalities might explain whether a linear relationship with the behavioural effect can be successfully captured.

With additional analyses more closely assessing the relationship between behavioural and neural effects, we however observed a correlation also in the visual modality, limited to the spatial-selectivity condition. This was observed within the same latency window showing a significant relationship in the auditory modality. Interestingly, although the latency window is the same, potentially suggesting a shared processing stage, the polarity of the effect is opposite. While in the auditory modality the behavioural effect increases for more negative polarities, in the visual modality it increases with positive polarities. This in turn suggests the involvement of different underlying sources of the effect, supporting the idea of modality-specific mechanisms. Since we did not find any significant relationship in the visual modality in the original linear regression analysis, this point however remains speculative.

Although so far what we observed is more consistent with at least partially independent sensory modality-specific mechanisms, the non-linear regression analysis showed instead a similarity between visual and auditory perception. Specifically, we observed a similar relationship between EEG activity across trials and the behavioural (binary) response provided by participants. Namely, the EEG response amplitude at around 300–500 ms can be predicted by the response that the participant will provide at the end of the trial. This is particularly interesting since the response will only be provided much later in the trial, and the reference itself did not provide sufficient information for decision-making. The serial dependence effect however can explain this relationship, since a biased reference representation can affect the choice. The similar timing of this signature in the visual and auditory modality might further suggest the involvement of a downstream decisional stage in brain areas involved with duration processing, such as the supplementary motor area (SMA) [55, 56]. Following the initial peak showing a similar timing in the visual and auditory condition, the relationship with participants’ choice continues to unfold differently in the two modalities, showing a subsequent (opposite) peak at around 600 ms in auditory perception but no such effect in vision. In all but one condition (i.e. visual spatial selectivity), this effect peaked at around the same time as the inducer effect in the non-linear analysis, supporting the idea that the effect of behavioural responses might be linked to serial dependence. Besides the influence that serial dependence has on the reference processing and its impact on the later decision, it is however possible that this relationship might reflect a more general perceptual decision-making process concerning the reference representation. Variability in the reference representation might indeed contribute to early decision processes occurring before the probe presentation and response selection, thus introducing a correlation that does not necessarily reflect serial dependence. Further evidence disentangling serial dependence from decision processes is thus needed to support our interpretation.

To summarise, our results show three main differences in the signatures of visual and auditory serial dependence. First, the double dissociation in the properties of the behavioural effect provides evidence that serial dependence is governed by different “rules”, consistent with the intrinsic properties of different modalities. Second, the modulation of ERPs as a function of the inducer shows different dynamics in the two modalities, with a more prominent peak before stimulus offset in vision, and a later, higher peak in auditory perception. Third, in the auditory condition, we observed a clear relationship between the behavioural and neural signature of perceptual history, which we were instead unable to capture in vision (at least with a linear regression analysis). This suggests a difference in how perceptual history is encoded in brain signals and how these signals relate to the behavioural effect. Using a non-linear regression model, on the other hand, we also highlighted a similarity between the two modalities: the relationship between EEG activity and choice in the task was observed in a broad latency window similar across modalities. Based on these results alone, it is difficult to confidently conclude that such a relationship stems from the exact same processing stage and neural generator, but its consistency across conditions at least suggests the possibility of a shared component, possibly related to a high-level representation of stimulus duration or perceptual decision-making.

A methodological limitation that we need to acknowledge, however, is that our EEG approach was largely explorative. Indeed, while we had hypotheses concerning the selectivity of the effects at the behavioural level, we did not have specific predictions when it comes to the difference in the neural signature in the two modalities. This in turn might have made us miss additional potential differences due to the choices made, like for instance basing the channel selection on the simple ERP difference between the inducer magnitudes. Only a few studies so far employed EEG to study serial dependence, and hence, it is difficult to come up with clear a priori hypotheses and predictions about where and when the signature of perceptual history might show the largest differences across modalities. Additionally, the choice of focusing on a small number of channels (two in each hemisphere) is likely another limiting factor. Brain activity reflecting serial dependence and its selectivity might indeed be more widespread or arise at different scalp locations. Further studies with different analytical approaches are thus needed to refine our findings and potentially demonstrate more robust differences across different sensory modalities. In terms of interpretation, while the results suggest differences in how perceptual history is implemented in the visual and auditory processing streams, this in principle does not completely exclude the existence of a more centralised, high-level serial dependence mechanism. The differences in the effect might indeed reflect differences in modality-specific processing that are unrelated to perceptual history and serial dependence, occurring earlier in the hierarchy and cascading to later processing stages. This possibility was considered in our initial design of the study, and this is the reason why we chose to use very salient modulations in terms of features and spatial positions. In doing so, any difference in the effect is less likely to arise from the different intrinsic sensitivity of each modality to the manipulations that could potentially limit the effect at a higher-level stage. Instead, it is more likely that the differences observed reflect specific properties of the serial dependence effect implemented in different modalities.

## Conclusions

Overall, our results provide evidence that perceptual history in time perception operates differently in different sensory modalities, suggesting the existence of at least partially independent mechanisms implemented within different sensory pathways. Our results converge with other studies that demonstrate a large variability in the properties of serial dependence when measured in different contexts and with different paradigms. Taken together, these results suggest that the serial dependence bias in different contexts might not reflect a unitary phenomenon, but different, independent phenomena with similar behavioural outcomes. In light of this, different frameworks of serial dependence based on perceptual or post-perceptual processes might not be mutually exclusive, but simply based on different types of effects emerging at different brain processing stages [31]. To conclude, our findings suggest that rather than being implemented in a centralised hub, serial dependence may stem from the same computational principle emerging independently along different brain processing pathways and at different stages.

## Methods

### Participants

A total of 64 participants took part in the study, with 20 subjects tested in Exp. 1 (14 females, age ± SD = 24.6 ± 2.4 years), and two groups of 25 subjects each tested in Exp. 2 (37 females, age ± SD = 25.1 ± 4.3 years). Note that the total number of participants is less than the sum of the individual conditions because a few participants were recruited in both experiments. Participants were all healthy volunteers, with normal or corrected-to-normal visual and auditory perception, and none reported any neurological, developmental, or psychiatric disorder. Since the experiment involved colour perception, we also asked participants to report any related condition (i.e. being colour blind). None of the participants reported any condition affecting colour perception. To have an exclusion criterion, we set a cut-off corresponding to a Weber’s fraction (WF) > 1. Two participants in the feature-selectivity condition of Exp. 2 were excluded from data analysis due to excessively poor performance exceeding this threshold (see *Behavioural data analysis*), while two participants were excluded from the spatial-selectivity condition of Exp. 2 due to equipment failure (i.e. EEG was not recorded properly). Prior to taking part in the experiment, all participants read and signed a written informed consent form. All experimental procedures were approved by the ethics committee of the International School for Advanced Studies (SISSA) and were in line with the declaration of Helsinki. The sample size of Exp. 1 was estimated based on the effect size (Cohen’s *d* from a *t*-test) of serial dependence in visual time perception observed in another study from our group (Togoli et al., in preparation). Namely, we computed an average estimate of the expected effect size based on previous data, which turned out to be *d* = 1.02. Assuming a two-tailed distribution and a power of 95%, the resulting minimum sample size was 15 subjects, which we rounded up to 20 to account for possible excluded participants. In Exp. 2, we based our estimate of effect size on another recent study from our group investigating the neural signature of perceptual history across different magnitude dimensions, including time [30]. The neural signature of serial dependence in time perception in this study was explored using a multivariate decoding procedure, which in the case of time perception showed an average effect size (Cohen’s *d*, again based on a *t*-test) of 0.8. Based on this effect size, a two-tailed distribution, and a power of 95%, we estimated a minimum sample of 19 subjects, which we conservatively increased to 25 to account for the possible exclusion of a few participants. None of the experiments reported here was pre-registered.

### Apparatus and stimuli

All the experiments were performed in a quiet and dimly lit room. Visual stimuli were presented on a 1920 × 1080 monitor screen running at 120 Hz, positioned at a distance of about 80 cm from the eyes of the participant. The auditory stimuli were presented through loudspeakers positioned behind the screen, at a distance of 50 cm from each other. Visual stimuli were circular noise textures that could either be composed of black and red squares or black and green squares (see below *Procedure*). All the stimuli were generated using the Psychophysics Toolbox [57, 58] on MATLAB (version r2021b; The Mathworks, Inc.). The circular area of the visual textures had a radius of 200 pixels (~ 3.4 degrees of visual angle, deg, from a viewing distance of 80 cm). The auditory stimuli had an intensity of ~ 65 dB measured from the position of the participant. In both Exp. 1 and Exp. 2, visual stimuli were presented either on the left or on the right of a central fixation point, centred on the middle horizontal plane of the screen, and with a horizontal eccentricity (from the centre of the stimulus to the centre of the screen) of 8.2 deg (480 pixels). Auditory stimuli were pure tones, defined by a frequency of either 700 Hz or 1100 Hz. The tones had a 2-ms ramp at the onset and offset to avoid sound distortions and clicks. In the “feature-selectivity” condition of Exp. 1 and Exp. 2, the tones were presented through both the speakers, while in the “spatial-selectivity” condition, the sounds were presented through only one of the speakers, i.e. either the right or the left one.

### Procedure

In Exp. 1, participants performed two different conditions in two different modalities, in a 2 × 2 within-subject design. The two conditions were the “feature-selectivity” condition, where we tested the selectivity of the serial dependence effect for the features of successive stimuli, and a “spatial-selectivity” condition, where we tested the selectivity of the effect for the position of successive stimuli.

Across all experiments, participants performed a duration discrimination task, comparing the duration of a constant reference stimulus (200 ms) against a variable probe with different durations randomised across trials (100, 140, 200, 280, or 400 ms). To induce serial dependence, we presented a task irrelevant “inducer” stimulus before the reference, which could be either 100 ms or 400 ms long, following a similar procedure already used in previous studies (e.g. [5, 7]). While inducer and reference stimuli could have different features or positions (see below), the reference and probe always had the same features and the same position. Each trial started with participants keeping their gaze on a central fixation point. First, the inducer stimulus was presented on the screen, followed by the reference after an inter-stimulus interval (ISI) of 250–350 ms, and finally the probe after an ISI of 350–450 ms. After the offset of the last stimulus, the fixation point turned red, signalling to the participants that the stimulus presentation was over and to provide a response. Participants were asked to compare the reference and the probe and to report which one between these two stimuli lasted longer in time, by pressing the appropriate key on a keyboard (either “2” or “3”, to indicate whether the second or third stimulus in the sequence was longer). After providing a response, the next trial started automatically after 400–500 ms. When explaining the task, the participants were told that the first stimulus in each sequence (i.e. the inducer) was always irrelevant to the task. In each sub-condition of the experiment (i.e. auditory feature-selectivity, auditory spatial-selectivity, visual feature-selectivity, and visual spatial-selectivity), each participant completed 5 blocks of 112 trials, for a total of 28 repetitions of each combination of inducer duration, inducer features/position (i.e. same/different features or same/different position), and probe stimulus duration. All the conditions were performed in a single day. Participants performed 5-10 practice trials before the start of the experiment, to ensure that they correctly understood the procedure, and never received feedback about their responses in the main experimental session.

In the feature-selectivity condition, we manipulated whether the features of inducer and reference were similar or different, intermixing “same” and “different” trials within the same blocks. In auditory perception, we chose to modulate the frequency (i.e. pitch) of the stimuli. Namely, while the pitch of reference and probe was always 700 Hz, the pitch of the inducer could be either 700 Hz (same features) or 1100 Hz (different features). In vision, we chose to modulate the colour of the stimuli. In this case, while the reference and probe stimuli were always black and red noise textures, the inducer could either be a black and red texture (same colour) or a black and green texture (different colour). Pitch and colour were chosen to define the features of the stimuli as they are easily and immediately discriminable, and hence, there is no uncertainty about whether the stimuli were the same or different. In this condition, all the stimuli were always presented in the same spatial position. Namely, auditory stimuli were presented through both the left and right speakers, while visual stimuli were presented either on the left or on the right of the central fixation point. In the spatial-selectivity condition, we instead manipulated the position of the stimuli and the spatial match of inducer and reference. In this case, all the auditory tones had a pitch of 700 Hz, and all the visual stimuli were black and red textures. In the auditory modality, the reference and probe were presented from either the left or the right speaker, randomised across trials. The inducer was thus presented either from the same speaker as the other stimuli (same position) or from the other speaker (different position). The same procedure was employed in vision. Namely, the reference and probe stimuli could be presented either on the left or on the right of the central fixation point (randomised across trials), and the inducer could be presented either in the same spatial position as the other stimuli (same position) or on the opposite portion of the screen (different position). The distance between different auditory sources (speakers) was 50 cm, while the distance between different positions of the visual stimuli (centre to centre) was ~16.4 degrees of visual angle (960 pixels). 

In Exp. 2, we used the same paradigm as in Exp. 1, with the addition of electroencephalographic (EEG) recording, but with a different experimental design. Namely, due to the longer time needed to carry out the EEG experiment, the feature-selectivity and spatial-selectivity conditions were performed by two independent groups of participants. Another difference compared to Exp. 1 was the ISI across the different stimuli, which was adapted to the EEG technique to ensure large enough epochs for the analysis (i.e. to have a sufficiently long blank epoch between the offset of the reference and the onset of the subsequent probe). Namely, the ISI between inducer and reference was 500–600 ms, while the ISI between reference and probe was 800–900 ms. The inter-trial interval was 300–400 ms. In Exp. 2, in each sub-condition, participants performed 5 blocks of 100 trials, for a total of 25 repetitions of each combination of inducer duration, features/position (same or different), and probe duration. Besides these differences, the procedure was identical to Exp. 1.

Additionally, in Exp. 2, we also performed a brief test of auditory spatial localisation before the main session of the auditory condition, in order to assess whether the spatial location of the sounds was discriminable. This test comprised a total of 60 trials. In each trial, a sound identical to the reference stimulus used in the main experiment (200 ms, 700 Hz) was presented from either the left or the right speaker, randomised in a trial-by-trial fashion (50%/50% proportion of left and right stimuli). After the presentation of the stimulus, participants were instructed to indicate the side (left or right) from which they heard the sound. After providing a response, the next trial started after a variable interval of 300–400 ms. The proportion of correct responses was then assessed to evaluate how well the location of the sounds could be discriminated.

### Behavioural data analysis

In both Exp. 1 and Exp. 2, we analysed the participants’ performance in the duration discrimination tasks to assess to what extent the perceived duration of the reference stimulus was affected by the preceding inducer, and whether this effect was modulated by the features or the relative spatial location of the stimuli. The proportion of responses in the task (“probe longer”) obtained from each participant in each condition and modality was fitted with a cumulative Gaussian (“psychometric”) function according to the maximum likelihood method [59]. In order to improve the psychometric fit, we applied a finger error rate correction (2.5%) to account for random errors and lapses of attention [60]. From the psychometric fit, we then computed the measures of accuracy and precision in the task. The “point of subjective equality” (PSE), which represents the perceived duration of the reference stimulus (i.e. accuracy), was defined as the median of the psychometric fit. To assess the precision in the task, we first computed the just noticeable difference (JND) as the difference in probe duration between the 50% and 75% “probe longer” response levels. Then, we computed the Weber’s fraction, which represents a measure of precision taking into account the perceived magnitude of the stimuli (WF = JND/PSE), which was used to assess the overall performance of the participants and exclude subjects showing insufficient performance. PSE, JND, and WF measures were computed separately for each level of inducer duration and condition. Based on the WF, two participants were excluded from the feature-selectivity condition of Exp. 2 as they showed excessively poor performance in the task (WF > 1).

To better assess and compare the serial dependence effects obtained in the different modalities as a function of the properties of the stimuli, we computed a “serial dependence effect” index based on the normalised difference between PSEs as a function of inducer duration, according to the following formula:

1 $$\mathrm{Serial}\;\mathrm{dependence}\;\mathrm{effect}\;=\;(({\mathrm{PSE}}_{400}-{\mathrm{PSE}}_{100})/{\mathrm{PSE}}_{100})\times100$$

where PSE_100_ refers to the PSE obtained when the reference was preceded by the shorter inducer (100 ms), and PSE_400_ refers to PSEs obtained with the longer inducer (400 ms). This index was calculated separately for each participant and condition (see Fig. 2). The differences in the serial dependence effect across modalities and conditions were assessed using a linear mixed-effect (LME) model regression, entering “modality” (visual vs. auditory), “condition” (feature vs. spatial selectivity), and “congruency” (same vs. different, either in terms of features or spatial position) as fixed effects, and the subjects as a random effect. This regression model was then followed up by a series of paired and one-sample *t*-tests to better characterise the pattern of effects. *T*-tests in the behavioural analysis were not corrected for multiple comparisons to ensure the sensitivity of the tests. The replication of the effects in two independent samples (Exp. 1 and Exp. 2) served instead as a way to ensure the robustness of the results.

### Electrophysiological recording and analysis

In Exp. 2, the electroencephalogram (EEG) was recorded throughout the experimental session, using the Biosemi ActiveTwo system and a 32-channel cap based on the 10–20 system layout. The EEG signals were recorded with a sampling rate of 2048 Hz. In addition to the 32 electrodes mounted on the cap, we also measured the electro-oculogram (EOG) via a channel attached below the left eye of the participant. The electrode offsets across channels were usually kept below 20 µV, but occasional values up to 30 µV were tolerated.

EEG pre-processing and data analysis was performed offline in MATLAB (version R2021b), using the EEGLAB [61] and ERPlab (63) packages. During pre-processing, EEG recordings were first high-pass filtered (0.1 Hz) and re-referenced to the average of all the channels used. The continuous EEG data were then segmented into epochs spanning from − 200 to 800 ms after stimulus onset, time-locking the epochs to the onset of the reference stimulus and sorting them as a function of the duration and properties of the preceding inducer. The pre-stimulus interval (− 200:0 ms) was used to perform baseline correction, in order to subtract any residual spurious effect related to the inducer stimulus itself from the response evoked by the reference. To clean up the data from noise and artifacts, we performed an independent component analysis (ICA). Furthermore, we applied a step-like artifact rejection procedure (amplitude threshold = 40 µV, window = 400 ms, step = 20 ms) aimed at further removing any remaining large artifact from the EEG signals, which yielded an average (± SD) rejection rate of 1% ± 1.87% of the trials. Finally, we applied a lowpass filter with a cut-off of 30 Hz before computing the ERPs.

To assess the neural signature of perceptual history, we performed a series of analyses on ERP and EEG data. First, as a measure of the effect of perceptual history, we computed the difference in ERP amplitude as a function of the duration of the inducer, irrespective of the properties of the stimuli, throughout all the EEG channels. The scalp topography of this measure was then used to select the channels of interest used for further analysis. To improve the signal-to-noise ratio, we selected two channels and used their average for further analyses (i.e. the channel showing peak contrast amplitude and an adjacent channel showing the highest amplitude among the surrounding electrodes). In the spatial-selectivity condition, we similarly selected the best channel and a nearby one, but this time considering the scalp locations contralateral to the position of the reference stimulus (i.e. two channels in the left hemisphere when the reference was presented on the right, and vice versa). In the feature selectivity condition, we selected T8 and P8 in the auditory modality, and P8 and PO4 in the visual modality. In the spatial-selectivity condition, we selected T8 and P8 in the right hemisphere and T7 and P7 in the left hemisphere, in both the auditory and visual conditions. This data-driven procedure for channel selection was implemented due to the lack of previous literature (especially in the auditory modality), which made it difficult to select the relevant channels a priori.

In our main ERP analysis, we assessed the contrast amplitude (i.e. difference in ERP amplitude as a function of inducer duration)—which was considered as a neural signature of the perceptual history effect induced by the inducer stimulus—separately for the different features or positions of the stimuli. First, to further increase the signal-to-noise ratio, we averaged the contrast amplitude across a series of 100-ms time windows, with a step of 25 ms. To compare the contrast amplitude across conditions where the inducer had either the same or different features or position, we performed a series of paired *t*-tests. To control for multiple comparisons, we used a cluster-based non-parametric test, which is described in detail below. Then, to assess the extent to which the contrast amplitude relates to the serial dependence bias measured behaviourally, we performed a linear mixed-effect regression analysis. Previous studies indeed show that this analysis can capture the relationship between the neural and behavioural signature of an effect, providing information about the brain processing stages that are relevant to behaviour [30, 60, 61]. In this analysis, we entered the difference in PSE corresponding to the 400-ms and 100-ms inducer (ΔPSE) as the dependent variable. As fixed effect factors, we entered the contrast amplitude (ΔERP), the match between inducer and reference (same vs. different, either features or position), and the interaction between these two factors. The subjects were added to the analysis as the random effect. This analysis was performed independently across each time window throughout the reference epoch. Again, multiple comparisons were controlled by performing a cluster-based non-parametric test.

Moreover, we also performed a non-linear regression analysis, considering the amplitude of EEG responses across individual trials rather than the averaged ERPs. The non-linear regression was chosen to gain a different perspective on the neural signature of serial dependence and the behavioural effect. Indeed, it allowed us to use the single-trial EEG and behavioural data instead of the aggregate, average measures, and thus, it is potentially more sensitive to the trial-by-trial modulations performed in our paradigm. In this analysis, we thus entered the trial-by-trial EEG amplitude as the dependent variable and assessed its relationship with the inducer and its properties, and the response that participants provided in each trial. In the feature-selectivity condition, we entered as factors the duration of the inducer (100 vs. 400 ms), the features of the inducer (same vs. different compared to the reference), and the behavioural response, i.e. the binary choice (0 or 1 corresponding to “reference longer” or “probe longer”; categorical variable) made by participants at the end of each trial. In the spatial-selectivity condition, we entered again the duration of the inducer and the behavioural response as factors, with the addition of the reference position (i.e. reference presented on the left or on the right of the fixation point) and the spatial match of the inducer (same position vs. different position). This non-linear analysis was used to assess the contribution of each of those factors to the amplitude of the trial-by-trial EEG response, which was characterised in terms of beta value. The beta value indicates the extent to which the EEG response changes (in terms of µV of amplitude) across the different levels of each factor, and hence, it could provide evidence for a relationship between the EEG activity, the experimental manipulations, and the participants’ response in each trial. Similarly to the linear regression analysis, the non-linear regression was performed independently at each time window across the reference epoch (100 ms, 25-ms step). Beta values across the group, at each time window, were then assessed using one-sample *t*-tests against the null hypothesis of zero effect. This series of tests was again controlled using a cluster-based non-parametric test.

Across the different tests performed during the ERP/EEG analysis, we controlled for multiple comparisons using a cluster size threshold and a cluster-based non-parametric test. First, to be considered as a potentially significant cluster, we set a threshold of at least three consecutive significant time windows from the original test performed (i.e. three consecutive significant paired *t*-tests, for example). This was done in line with previous studies from our group ([30, 61]) in order to make the non-parametric approach more conservative, considering the typical high sensitivity of this method (e.g. [46]). The choice of having at least three significant consecutive tests was based on the width of the latency windows used in the tests (100 ms, 25-ms step), so that the last window in a significant cluster overlapped with the first one no more than 50%. In the non-parametric test, each cluster of consecutive time windows was compared to the clusters that could be obtained across several repetitions of the same test but with either randomised data or by performing permutations of the data being compared. Regarding the cluster-based procedure in the case of the paired *t*-tests, we split the data corresponding to the two conditions being compared into two randomly selected subsets, and created two new sets of data by concatenating two halves corresponding to the two original conditions. We then performed the paired *t*-test across these intermixed datasets. This procedure was performed across each time window within each of the clusters observed in the actual analysis, and repeated 10,000 times. To test for the significance of the actual cluster, we set a cluster-level threshold equal to the minimum *t*-value observed in the actual cluster, and assessed how many consecutive time windows exceeded this threshold within the simulated cluster. We then counted how many times the simulation yielded a cluster of consecutive time windows equal to the actual cluster, and defined the cluster-level *p*-value as the proportion of times that the same number of consecutive time windows was observed across the 10,000 simulations. In the case of the linear regression analysis, the procedure was identical, but to compute the simulated clusters we randomly shuffled the design matrix containing the values of the predictors. Finally, in the case of the one-sample t-tests used to assess the significance of beta values from the non-linear analysis, we flipped the sign of a random half of data before performing the one-sample *t*-test. All regression analyses were performed in MATLAB (version r2021b).

## Data Availability

All the data generated during the experiments described in this manuscript and the experimental code is freely available on Open Science Framework at this link: https://osf.io/7ex9y/ (DOI: 10.17605/OSF.IO/7EX9Y). (DOI: 10.17605/OSF.IO/7EX9Y).

## Abbreviations

EEG: Electroencephalography
ERP: Event-related potential
LME: Linear mixed-effect model
PSE: Point of subjective equality
JND: Just noticeable difference
WF: Weber’s fraction
